# Multicentric Castleman’s disease with voltage-gated potassium channel antibody-positive limbic encephalitis: a case report

**DOI:** 10.1186/s12883-015-0266-8

**Published:** 2015-02-04

**Authors:** Vikram R Rao, Leland E Lim, Dean Fong, Nina I Garga, Karen L Parko

**Affiliations:** Department of Neurology, University of California, San Francisco, 400 Parnassus Ave, 8th Floor, San Francisco, CA 94143 USA; Neurology Service, Department of Veteran Affairs, Palo Alto Health Care System, 3801 Miranda Avenue, Palo Alto, CA 94304 USA; Department of Neurology and Neurological Sciences, Stanford University School of Medicine, Stanford, CA 94305 USA; Pathology and Laboratory Medicine Service, Department of Veteran Affairs, Palo Alto Health Care System (113), 3801 Miranda Avenue, Palo Alto, CA 94304 USA; Epilepsy Center of Excellence, San Francisco Veteran Affairs Medical Center, 4150 Clement Street, Box 127E, San Francisco, CA 94121 USA

**Keywords:** Castleman’s disease, Lymphoproliferative, Limbic encephalitis, Voltage-gated potassium channel, VGKC, Paraneoplastic, Seizure

## Abstract

**Background:**

Castleman’s disease is a rare lymphoproliferative disorder which occurs in localized and multicentric forms and can mimic lymphoma. Despite its well-known association with certain autoimmune diseases, including paraneoplastic pemphigus and myasthenia gravis, Castleman’s disease has not previously been associated with limbic encephalitis.

**Case presentation:**

We report the case of a 47-year old Caucasian man who presented with subacute onset of constitutional symptoms, diffuse lymphadenopathy, and stereotyped spells involving olfactory aura, nausea, disorientation, and unresponsiveness. He was found to have focal dyscognitive seizures of temporal lobe origin, cerebrospinal fluid with lymphocytic pleocytosis, hyponatremia, and serum positive for voltage-gated potassium channel antibodies, consistent with limbic encephalitis. An extensive infectious workup was unrevealing, but lymph node biopsy revealed multicentric Castleman’s disease. His symptoms improved with antiepileptic drugs and immunotherapy.

**Conclusion:**

This case highlights the clinical diversity of voltage-gated potassium channel autoimmunity and expands the association of Castleman’s disease and autoimmune syndromes to include limbic encephalitis. Clinicians should be aware that paraneoplastic disorders of the central nervous system can be related to underlying hematologic disorders such as Castleman’s disease.

## Background

Castleman’s disease (CD) is a rare lymphoproliferative disorder characterized by enlarged, hyperplastic lymph nodes that can mimic lymphoma. First described in 1954 [1], CD occurs in several histologic variants, including hyaline-vascular, plasma cell, and mixed types. Two clinical presentations can be distinguished: a localized or unicentric form, which is typically indolent and treated with local excision, and a systemic or multicentric form (MCD), which usually involves the plasma cell variant and portends a worse prognosis. MCD presents with diffuse lymphadenopathy, hepatosplenomegaly, fevers, night sweats, and fatigue. The pathogenesis of MCD is thought to be driven by human herpesvirus 8 (HHV8), which latently infects B cells in healthy individuals but can reactivate in the setting of immune system compromise, such as human immunodeficiency virus (HIV) infection. Reactivated HHV8 promotes production of interleukin-6 (IL-6), which stimulates B cell proliferation and lymphoid hyperplasia, and treatment of MCD can be directed at lowering levels of this cytokine [2].

CD most commonly presents with lymphadenopathy in the neck, chest, and/or abdomen. Central nervous system (CNS) involvement is rare, but intracranial, extra-axial lesions mimicking meningiomas have been reported in CD and can present with seizures [3,4], presumably due to their direct impingement on cortex. CD has been associated with several autoimmune diseases [5], including paraneoplastic pemphigus, systemic lupus erythematosus, rheumatoid arthritis, myasthenia gravis, and polyneuropathy, organomegaly, endocrinopathy, monoclonal gammopathy, and skin changes (POEMS) syndrome, but only a single report describes a paraneoplastic CNS syndrome in the setting of CD [6]. Limbic encephalitis (LE), a classic CNS autoimmune disorder, presents with cognitive changes and focal seizures of mesial temporal lobe origin. LE can occur with or without identifiable underlying malignancy and can be seronegative or associated with specific antibodies. Malignant hemopathies—including leukemia [7] and, more commonly, lymphomas [8]—have been associated with paraneoplastic LE, but, to our knowledge, CD has not.

Here we report the case of a patient who presented with subacute onset of constitutional symptoms and seizures and was found to have MCD, LE, and voltage-gated potassium channel (VGKC) antibodies. This case illustrates novel linkage of two rare clinical entities through an antibody well-known to mediate paraneoplastic neurological syndromes.

## Case presentation

**Figure 1:**
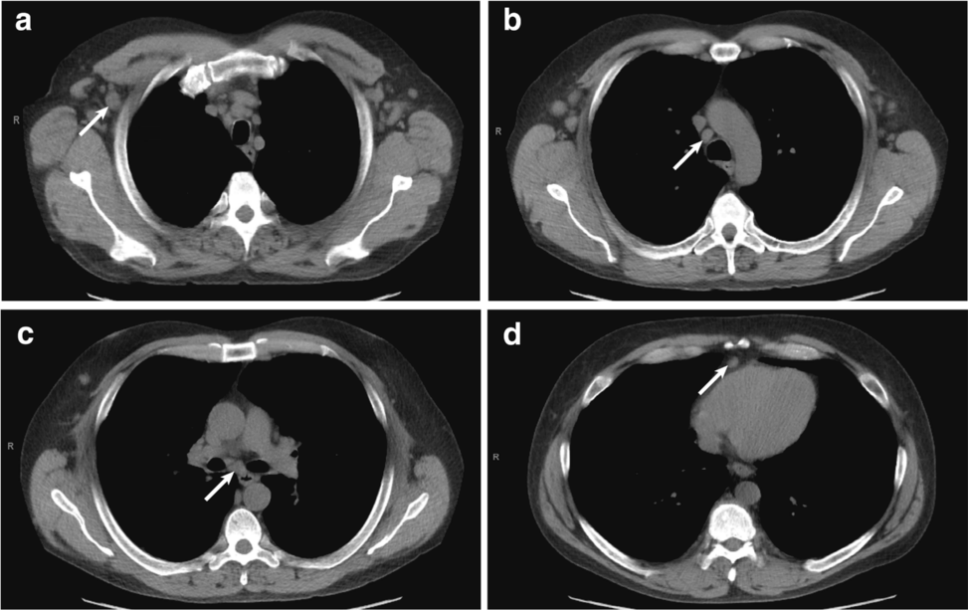
**Lymphadenopathy on chest CT.** Chest CT demonstrating enlarged lymph nodes (arrows) involving the right axillary **(a)**, paratracheal **(b)**, subcarinal **(c)**, and cardiophrenic **(d)** regions.

A 47-year old Caucasian man with history of depression and remote polysubstance abuse presented to his primary care physician with two months of cough, dyspnea, and asthenia. Chest radiography demonstrated patchy, diffuse interstitial infiltrates. Chest computed tomography (CT) disclosed diffuse lymphadenopathy, splenomegaly, and bilateral bronchiectatic changes (Figure 1). Body positron emission tomography (PET)/CT showed diffuse, hypermetabolic (mild-moderate ^18^ F-fluorodeoxyglucose (FDG) uptake) lymphadenopathy without other evidence of malignancy. The initial differential diagnosis included atypical respiratory infection, granulomatous disease, and lymphoproliferative disorder. He underwent bronchoscopy with bronchoalveolar lavage, with negative staining for acid-fast bacilli, no malignant cells on cytology, and no growth in bacterial and fungal cultures. Transbronchial lung biopsy revealed non-specific chronic inflammation, and fine needle aspirate of a left neck lymph node was non-diagnostic. A repeat bronchoscopic evaluation nine months later was similarly unrevealing. Serum protein electrophoresis showed no evidence of monoclonal gammopathy, and angiotensin-converting enzyme levels were within normal limits. Serum inflammatory markers were elevated (erythrocyte sedimentation rate 111 mm/hr, C-reactive protein 7.28 mg/dL), but an extensive biochemical evaluation for infectious etiologies, including HIV, tuberculosis, coccidioidomycosis, aspergillosis, cryptococcosis, blastomycosis, and histoplasmosis, was negative. Due to an elevated *Strongyloides* IgG antibody titer, he was treated with ivermectin but had no improvement in his symptoms.

While this workup was in progress, the patient described progressive memory difficulty and recurrent, stereotyped spells involving perception of an unpleasant odor followed by nausea and dizziness. The spells lasted about one minute, occurred up to seven times per day, and coincided with episodic confusion noticed by his girlfriend. A routine electroencephalogram (EEG) was normal, and the patient was treated empirically with levetiracetam without benefit.

**Figure 2:**
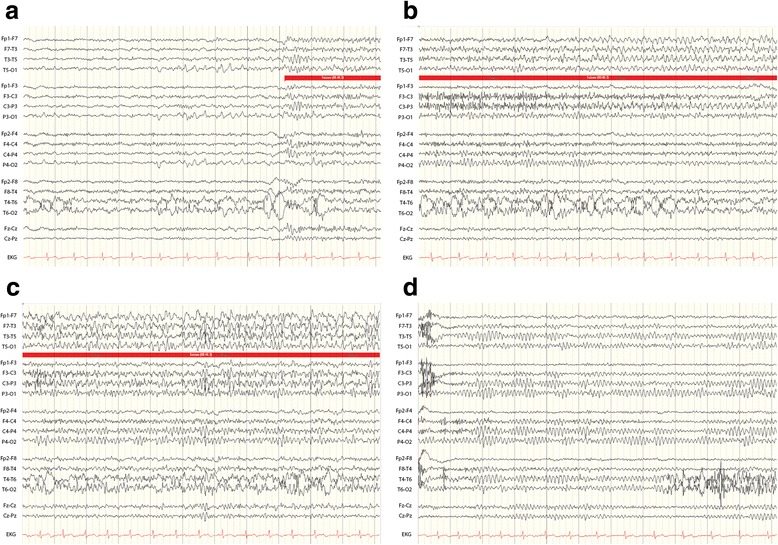
**Ictal EEG.** EEG during a seizure (red bar) arising from light sleep. Initially, there is emergence of theta/alpha range frequencies broadly over the left temporal region, maximal at electrodes F7 and T3 **(a)**. Left temporal waveforms become rhythmic **(b)** and evolve in frequency and amplitude while acquiring sharpened elements **(c)** before return to interictal background **(d)**. Clinically, the patient was observed to have lip-smacking automatisms during this seizure.

His neurological exam was notable for short-term memory impairment (1 out of 3 object recall after 5-minute delay) and hyporeflexia with length-dependent sensory loss in the lower extremities. Routine bloodwork revealed hyponatremia, with serum sodium of 131 mmol/L. Lumbar puncture was performed, and cerebrospinal fluid (CSF) analysis revealed glucose 55 mg/dL, total protein 33 mg/dL, and 12/mm^3^ white blood cells (88% lymphocytes, 11% monocytes) and 15/mm^3^ red blood cells. CSF cytology was negative. Gadolinium-enhanced brain magnetic resonance imaging (MRI) at 3.0 Tesla was normal. Video-EEG monitoring performed over two days captured twelve electrographic seizures—most were subclinical but some had associated dyscognitive features—with ictal onset over the left temporal region (Figure 2). Electrographic features of the seizures, including early emergence of rhythmic theta- and alpha-range frequencies, suggested mesial temporal origin. Levetiracetam was substituted with divalproex, which led to complete resolution of the patient’s seizures based both on his report and on repeat video-EEG monitoring one month later. His cognitive deficits also improved subjectively, though formal neuropsychological testing was not pursued.

The constellation of subacute encephalopathy, temporal lobe seizures, and mild lymphocytic CSF pleocytosis raised the possibility of autoimmune encephalitis. Serological analysis revealed elevated voltage-gated potassium channel (VGKC) antibodies (850 pmol/L; Athena Diagnostics, reference range 31–88 pmol/L). Testing for *N*-methyl-D-aspartate receptor and glutamic acid decarboxylase antibodies was negative. VGKC antibodies can be associated with underlying neoplastic disease, and, to further evaluate this possibility, excisional biopsy of a level V lymph node in the right neck was performed.

**Figure 3:**
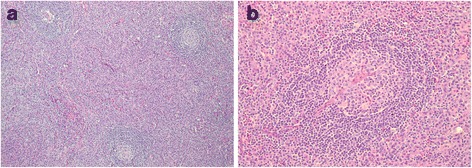
**Lymph node biopsy pathology.** 10x **(a)** and 20x **(b)** images of lymph node tissue sections were captured on an Olympus BX40 microscope with Olympus SC100 camera and Olympus analySIS getIT software. **(a)** Atrophic germinal centers with mantle zone lymphocytes arranged in concentric rings create an “onion-skinning” appearance. Note that the interfollicular areas are expanded by sheets of plasma cells. **(b)** Germinal center with penetrating vessels and a “lollipop” appearance.

Histologic sections revealed lymph nodes with variable sized follicles, some with atrophic germinal centers (Figure 3a). Germinal centers with penetrating vessels were present, conferring a “lollipop” appearance (Figure 3b). Mantle zone lymphocytes were arranged in concentric rings (“onion-skinning”). The interfollicular areas and medulla were expanded by large sheets of mature plasma cells. No granulomas were seen. Immunohistochemistry demonstrated the presence of polytypic plasma cells. Flow cytometry provided no evidence for a clonal lymphocyte population or aberrant antigen expression. IL-6 concentration in undiluted serum was 7.12 pg/mL (reference range 0.31-5.00 pg/mL) but the level was 174.08 pg/mL when tested in serial dilutions, suggesting prozone effect. Taken together, these results were most consistent with MCD, plasma cell variant. Of note, HHV8 staining of lymphoid tissue and serum HHV8 immunofluorescence assay were both negative. Repeat HIV antibody testing was also negative.

The patient was treated with two four-week cycles of weekly rituximab infusions followed by maintenance rituximab infusions every two months for a planned two-year course. Antiepileptic drug therapy with divalproex was continued with some dose adjustment required for breakthrough seizures. Serum IL-6 levels were not monitored to track response to therapy, but serial PET scans have shown decreased size and FDG-avidity of diffuse lymphadenopathy without complete resolution. A repeat VGKC antibody titer six months after initiation of rituximab was lower but remained elevated at 656 pmol/L. At last follow-up, the patient’s cough was persistent, now being managed with prednisone, but the constitutional symptoms that led to initial presentation had resolved.

## Discussion

This case illustrates the typical features of MCD: onset in middle age, non-specific constitutional symptoms suggestive of inflammatory disease, diffuse lymphadenopathy and hepatosplenomegaly mimicking lymphoma, and, often, cough or dyspnea, which is more common in HIV-positive patients and may relate to a non-infectious pulmonary interstitial lymphocytic infiltrate. Mild sensory polyneuropathy, as found in our patient, can occur in 20% of patients with MCD [9]. Manifestations of MCD are thus non-specific and diagnosis is made by histopathology, which most commonly reveals the plasma cell variant. Although HHV8 is central to the pathogenesis of HIV-positive CD, its role in HIV-negative disease remains controversial [9] because HHV8 is found in only 50% of these patients [10]. Our patient was found to be HIV-negative and HHV8-negative, which may represent a nosologically-distinct disease entity, termed idiopathic MCD, whose pathogenesis is speculated to involve autoimmune mechanisms [11]. Thus, this case raises the intriguing possibility that VGKC antibodies might be important for the pathogenesis of certain forms of MCD.

Our patient also developed cardinal features of LE associated with VGKC antibodies, including subacute encephalopathy, temporal lobe seizures, CSF lymphocytic pleocytosis, and hyponatremia [12]. Brain MRI was normal, as is the case in up to 45% of patients with LE and VGKC antibodies [13]. VGKC antibodies are thought to be pathogenic in LE, but the antibodies are actually directed against epitopes in the extracellular domains of proteins tightly complexed with VGKCs *in vivo*; the most common target in LE is the leucine-rich glioma-inactivated 1 (LGI-1) protein [12]. Although we did not test specifically for this immunoreactivity, our patient did not demonstrate the faciobrachial dystonic seizure semiology that often precedes onset of LE in LGI-1 antibody-positive patients [14].

Previous reports have described patients with intracranial CD lesions presenting with seizures [3,4], presumably due to mass effect and irritation of adjacent cortex. CD is also known to be associated with paraneoplastic syndromes affecting the peripheral nervous system (PNS), including myasthenia gravis and POEMS syndrome [5]. One reported case of cerebellar degeneration in the setting of CD may represent an autoimmune CNS syndrome, but no antibody was identified [6]. Only 30% of patients with antibodies against VGKC have tumors, but VGKC antibodies have been reported in association with thymoma and lung cancer [15]. To our knowledge, this is the first report of CD associated with either LE or VGKC antibodies.

LE associated with VGKC antibodies is frequently responsive to immunotherapy, and reduction in antibody titer correlates with clinical improvement [16]. Our patient’s seizures were relatively easily controlled with antiepileptic drug monotherapy, though we cannot exclude the possibility that steroids used later for pulmonary indications also contributed to seizure control. Rituximab was initiated primarily for treatment of MCD. That only modest decrease in VGKC antibody titer was seen following treatment with rituximab, however, may indicate that paraneoplastic LE in the setting of MCD is more treatment-resistant than other forms of LE. Three-year disease-free survival in HIV-negative patients with plasma cell variant MCD is 45% [17], so our patient’s prognosis remains guarded. Other treatment options, including the anti-IL-6 monoclonal antibody, siltuximab [2], are being considered.

The range of autoimmune disorders associated with CD is broader than previously known, and VGKC antibodies may play an important pathogenic role. In addition to paraneoplastic syndromes affecting the PNS, clinicians should be aware that CD can be associated with LE and other CNS manifestations even in the absence of overt intracranial disease.

## Conclusions

Castleman’s disease can be associated with voltage-gated potassium channel antibodies and paraneoplastic syndromes involving the central nervous system. Although rare, Castleman’s disease should be considered in patients presenting with features of limbic encephalitis and evidence of an underlying lymphoproliferative disorder.

## Consent

Written informed consent was obtained from the patient for publication of this case report and accompanying images. A copy of the written consent is available for review by the Editor-in-Chief of this journal.
